# The Effect of Household Technology on Child Health: Evidence from China’s “Home Appliances Going to the Countryside” Policy

**DOI:** 10.3390/ijerph191911976

**Published:** 2022-09-22

**Authors:** Junhui Shi, Fang Wang, Huan Wang

**Affiliations:** College of Management, Sichuan Agricultural University, Chengdu 611130, China

**Keywords:** child health, household technology, HAGC policy, HAZ score, WAZ score, BAZ score

## Abstract

This paper examined the effects of household technology on child health using evidence from the Chinese government’s “Home Appliances Going to the Countryside” policy. A difference-in-differences approach was employed to examine 2000 to 2015 data from the China Health and Nutrition Survey data from before the policy in 2007 to after the policy was implemented. It was found that the policy-induced household technology adoption significantly increased child health, especially girls’ health. Various sensitivity tests proved this finding to be robust. The potential paths through which household technology improved child health were also examined from which it was found that parental care for children and increased nutrition were effective paths between household technology and health status. These results could guide policymakers when constructing and developing a supportive child health system in China.

## 1. Introduction

Technology helps to leverage and maintain social, physical, emotional, intellectual, and spiritual well-being of children, in an environment where children are co-engaged with an adult [1]. The interdependence between household technology (refrigerators, washing machines, color televisions, etc.) and human capital has been widely examined in the social sciences [2,3,4,5]. As an important determinant of human capital, health has been seen to be most directly affected by home technology [2]. Research has shown that childhood health conditions can have a lasting impact on adult health and socioeconomic status [6,7]. However, there have been few studies examining the effects of household technology on child health, especially in developing nations.

Despite the global efforts to reduce childhood mortality, it has been estimated there will be about 10 million child deaths between 2019 and 2030 United Nations International Children’s Emergency Fund (UNICEF) (2021): Levels and Trends in Child Mortality. United Nations Inter-Agency Group for Child Mortality Estimation (UN IGME), Report 2021 from https://data.unicef.org/resources/levels-and-trends-in-child-mortality/ (accessed on 28 May 2022). Malnutrition is an underlying determinant for 45% of all annual childhood deaths [8]. Worldwide, over 200 million children are undernourished, especially in developing countries where the child malnutrition rate is higher [9]. As children’s health is directly related to the future quality of the labor force and the population, investing in children’s health means investing in national health [10]. Therefore, child health has been on the public policy agenda of many developed and developing countries. As an essential household asset, durable appliances can decrease the time children spend working in the home and therefore, provide greater opportunities for capital investment in child activities [11]. Some home appliances such as refrigerators can improve children’s nutritional outcomes [12]. As China is one of the most populous developing countries and has had rapid economic development, it is of particular interest to examine the relationship between Chinese children’s health status and the availability of household technology.

Identifying the impact of household technology on child health typically has endogeneity problems because many factors influence a household’s ability to adopt appliances and children’s health. For example, high-income households may be more likely to adopt household technologies and the children are more likely to have better health. Fortunately, the Chinese government’s “Home Appliances Going to the Countryside” (HAGC) policy can avoid these possible endogeneity issues. The HAGC policy started in 2007 and offered rural households in certain provinces 13% subsidies on specific durable goods’ categories. In 2009, all rural regions in mainland China became eligible for the subsidies. The HAGC policy is a national strategic plan; therefore, it could not be independently controlled in each region. Therefore, the qualification for HAGC subsidies could be regarded as a quasi-natural experiment to accurately examine the causal effects of household technology on child health by comparing the health status of rural and urban children before and after the HAGC policy implementation.

This study, therefore, sought to clarify the relationship between household technology and individual health outcomes in China. As health investment in children is more effective than in adults, this study used a subpopulation of children to investigate the relationship. Data from the China Health and Nutrition Survey (CHNS) were examined and a difference-in-differences (DID) model was employed to estimate the causal effects between household technology and child health.

This paper deepens the understanding of the household technology adoption effect mechanism on child health and the sustainable development of human capital in developing countries. This study also examined the heterogeneous impacts of household technology on different genders and ages; and to cater to the possible child health differences between sampled urban and rural populations propensity score matching was combined with the difference-in-differences approach (PSM-DID) to estimate the effects of household technology on child health. The PSM-DID approach was able to compare the health status of children in the pilot and non-pilot areas before and after the HAGC policy implementation, which ensured the robustness of the benchmark model estimation results.

The remainder of this paper is organized as follows: Section 2 presents an introduction to China’s “Home Appliances Going to the Countryside” policy and discusses the possible household technology effect mechanisms on child health: Section 3 describes the data, the research methods, and the variables used for the empirical analyses: Section 4 presents the results and discussion: and Section 5 provides the research conclusions.

## 2. Background

### 2.1. China’s “Home Appliances Going to the Countryside” Policy

Under the shadow of the global financial crisis, at the end of 2007, the Chinese government launched the “Home Appliances Going to the Countryside” policy to stabilize the domestic economy and improve the living standards of rural residents. The HAGC policy was the earliest, most influential, and longest-lasting policy to stimulate the consumption of durable household goods. In December 2007, the Chinese government provided HAGC financial subsidies to three pilot provinces: Shandong, Henan, and Sichuan. The HAGC policy gave rural purchasers of selected home appliances a 13% subsidy. In December 2008, the program was extended to 10 other provinces: Inner Mongolia, Liaoning, Dalian, Heilongjiang, Anhui, Hubei, Hunan, Guangxi, Chongqin, and Shanxi. In 2009, the 13% subsidies were extended to all households in mainland China The implementation plan for the pilot work of household appliances to the countryside, from the Ministry of Commerce and Ministry of finance of the people’s Republic of China (http://www.mofcom.gov.cn/article/h/redht/200712/20071205303046.shtml (accessed on 28 May 2022)), was applied with agricultural residence registrations, which were calculated based on the export tax rebate rate. The subsidy funds were jointly paid by the central and local governments, of which 80% were borne by central finance and 20% by local finance. During the policy implementation period and according to the agreement, the subsidized products included color TV sets, refrigerators, and washing machines, which were produced by the bid-winning enterprises. A total of 1264 models of the appliances qualified, which were then branded with the unique HAGC logo. The price ceilings for these products were CNY3500 for color TVs, CNY2500 for refrigerators, and CNY2000 for washing machines. By the end of September 2012, China had sold 275 million home appliance sets to rural customers, had achieved sales revenue of 690.91 billion CNY (103.65 billion USD), and had issued HAGC subsidies worth 80.12 billion CNY (12.02 billion USD) (China government website, Ministry of Commerce: 6.97 million sets of home appliances sold to the countryside in February, http://www.gov.cn/govweb/banshi/2012-03/08/content_2086268.htm (accessed on 28 May 2022)).

Previous studies have identified that color TVs, washing machines, and refrigerators can have a significant impact on health [2]. Therefore, this paper only considered these three home appliances subsidized by the HAGC policy and did not consider other products, such as motorcycles, computers, water heaters, or air conditioners, all of which were also subsidized from 2009. These three HAGC subsidized household appliances were chosen because they can affect family time and nutrition, as explained in our theoretical framework. For example, washing machines can reduce the time needed to hand wash clothes, and therefore reduce the time needed for housework and increase the time available for child-care. Similarly, having a refrigerator means that households do not need to shop as often, can more efficiently produce food for home consumption [13], and can improve general nutrition and health.

**Figure 1:**
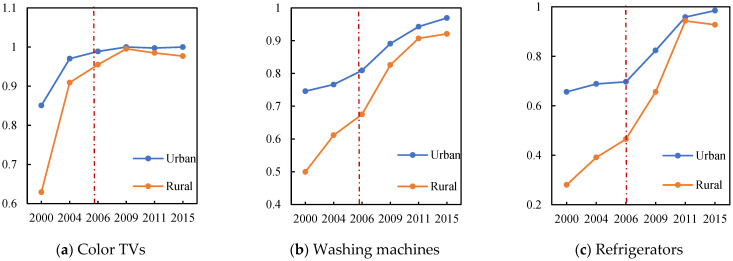
Adoption rate for household appliances before and after the HAGC policy implementation.

The HAGC policy had a significant impact on the household appliance adoption rate. Figure 1 shows the changes in the household appliance adoption rate in rural and urban areas before and after the HAGC policy implementation. After the policy was implemented, the differences in the household appliance adoption rates between the rural and urban areas decreased significantly, which indicated that the HAGC policy had had an exogenous impact on home technology adoption. Therefore, the HAGC policy implementation could be regarded as a quasi-natural experiment, with the policy-affected rural children being the treatment group, and the urban children not affected by the policy being the control group. The health status differences between the treatment group and the control group before and after the policy implementation were compared to determine the household technology impact on child health.

### 2.2. Conceptual Framework

The household technology effect mechanisms on child health are complex. First, household appliance adoption affects personal time allocation. Here, the specific focus was on the time allocated by parents to take care of their children and do the housework. Household appliances have been proven to decrease the time needed for housework, which means that parents can allocate more time to taking care of their children, which should result in improved child health. Second, household appliance adoption can improve nutrition. For example, with a refrigerator, fresh food such as meat and vegetables can be stored at home, which would then improve nutritional intake. Not having to shop every day would also free up time for the preparation of more nutritious meals, which could also improve child health.

**Figure 2:**
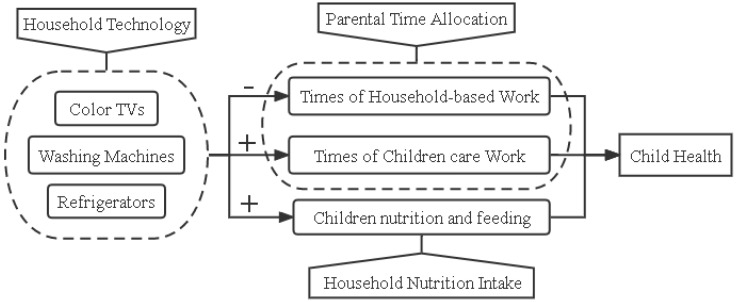
How household technology affects child health.

Household time allocation has been widely discussed in previous studies. Becker first proposed a time allocation theory and a more recent study incorporated time-saving appliance ownership into Becker’s model [14]. Bowden and Offer claimed that time-saving appliances in home appliances, such as refrigerators, microwave ovens, and washing machines, could reduce the time required to perform household work. When a household adopts time-saving home appliances, family members can reallocate time to other activities, such as leisure and schooling. Tewari and Wang and Coen Ptani, et al. found that home appliance adoption reduced the time required to perform household work and allowed more women to enter the labor force [3,13], and other studies have analyzed the allocation of parent and child time in a broader family context [15]. However, few studies have examined the effect of household technologies on child health outcomes. Using data from China, Kerr found that household appliance ownership had a positive impact on child school enrollment rates and decreased child labor participation rates [4]. However, studies on the time-saving effect of home appliances have not addressed child health outcomes. Therefore, based on Becker’s time allocation theory, this paper proposes a “household technology adoption-parental time allocation-child health” framework. As Figure 2 shows, household appliance adoption can change parental time allocations, which can relieve parental pressure and have a significant influence on children [16]. Parental time investment has been found to play a crucial role in fostering child development, such as health and skills [17]. It was assumed in this paper that household appliance adoption would reduce housework and increase the time that parents spend with their children, which in turn would improve the children’s health.

The adoption of household appliances can also improve the standards of cleanliness and meal nutrition for the home and for clothes, which could improve child health [18]. Heard et al. found that refrigerator ownership significantly increased nutrient intake [19], and other studies on child health found that food storage and preparation equipment was linked to child health [5] because refrigerators allowed families to introduce more high-protein foods into their diets [20] and reduced food contamination [21]. Research has also confirmed that washing machines remove bacteria from clothes and bedding and promote human health [22]. As Figure 1 shows, household appliance adoption can directly affect food processing nutrition and positively affect child health. Therefore, based on previous research, a “household technology adoption-family nutrition intake-child health” framework is proposed that assumes that the adoption of household appliances increases child nutrition and health.

Whether home appliance adoption affects child health conditions through either a change in parental time allocation or household nutrient intake was the empirical question. Data from many developed countries have found that household appliance adoption is a slow, long-term process [23], and there is little comprehensive data on whether improvements in household technology increase child health [24]. Therefore, to accurately explore the effect of household appliances on child health, the exogenous impact of the HAGC policy was examined.

## 3. Data, Variables, and Method

### 3.1. Data

**Figure 3:**
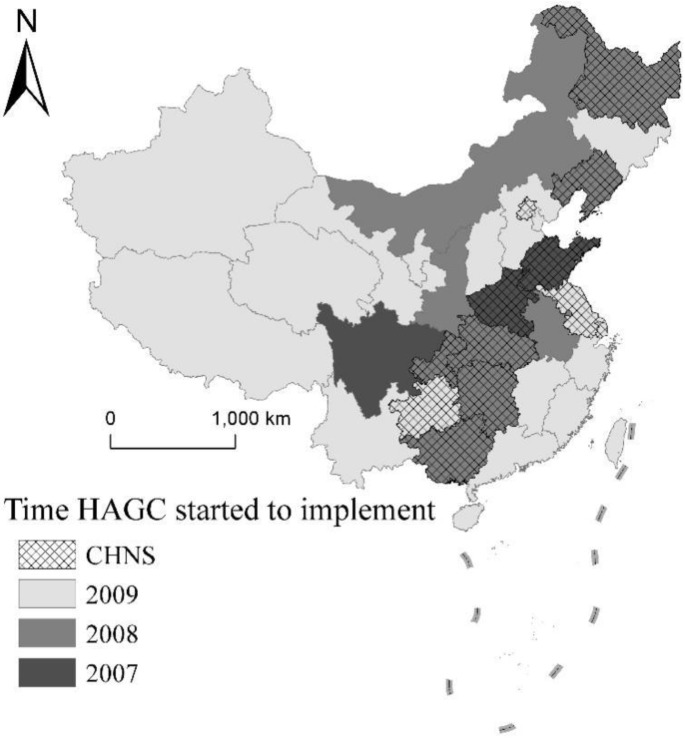
Implementation of the China HAGC by year across provinces and regions of CHNS.

The data were drawn from the China Health and Nutrition Survey (CHNS). CHNS is an ongoing longitudinal data project launched collaboratively by the Carolina Population Center at the University of North Carolina at Chapel Hill and the National Institute of Nutrition and Food Safety at the Chinese Center for Disease Control and Prevention. (https://www.cpc.unc.edu/projects/china (accessed on 28 May 2022)). This project examined the effects of the health, nutrition, and family planning policies implemented by national, and local governments during China’s social and economic transformation on population health and nutrition. The CHNS data included health status, time allocation, nutritional intake at the individual level, and appliance adoption at the household level. The CHNS used a multistage, random clustering process to draw the sample, with data being collected in 12 provinces: Guangxi, Guizhou, Henan, Hubei, Hunan, Jiangsu, Liaoning and Shandong, Heilongjiang, Beijing, Shanghai, and Chongqing. Out of these twelve provinces, the HAGC policy was introduced in rural areas: in Henan and Shandong in December 2007; in Guangxi, Heilongjiang, Hubei, Hunan, and Liaoning in December 2008; and in Guizhou and Jiangsu in February 2009.; from the same households in 1989, 1991, 1993, 1997, 2000, 2004, 2006, 2009, 2011 and 2015. The data employed in this study were collected in 2000, 2004, 2006, 2009, 2011, and 2015. As the HAGC policy was implemented in 2007, the years 2000, 2004, and 2006 were taken as the “pre-policy” periods and the years 2009, 2011, and 2015 were taken as the “post-policy” periods. Data from 2000 were chosen because before 2000, China had had major national reforms that may have impacted the household decisions to adopt household appliances. The Chinese government provided HAGC financial subsidies to Shandong, Henan, and Sichuan in December 2007, therefore, the policy effect was not evident until 2008. In addition, the 2009 CHNS was conducted in July and August 2009 and reflected the situation in 2008. The 2009 CHNS survey data only reflected the HAGC policy implementation in the pilot provinces (Shandong and Henan). In order to allow readers to clearly understand the distribution of HAGC policy implementation and CHNS survey data, we draw on previous studies [25] to create Figure 3.

In this paper, children are defined as those younger than 18 years old; therefore, the baseline sample was children between 0 and 18 years old. When the children were under six years old, the respective parents completed the questionnaire on their behalf. Data from respondents who reported zero or missing health outcomes or failed to report the parental time use or household nutrition information were removed; therefore, the final sample was a panel of 5085 children.

### 3.2. Variables

#### 3.2.1. Dependent Variables

As in previous studies, the dependent variable health outcomes in this study were ‘height-for-age Z-score (HAZ),’ ‘weight-for-age Z-score (WAZ)’, and ‘BMI-for-age Z-score (BAZ)’. HAZ, WAZ, and BAZ are common measures for assessing childhood malnutrition and have been extensively [26] adopted as child health measures [11,27]. The HAZ, WAZ, and BAZ were determined using Equations (1), (2), and (3), respectively.

(1) $${\mathrm{HAZ}}_{\mathrm{ij}}=\frac{{\;\mathrm{height}}_{\mathrm{ij}}-M{\left(t\right)}_{j}}{\mathrm{Std}.{\mathrm{Dev}}_{j}}$$

(2) $${\mathrm{WAZ}}_{\mathrm{ij}}=\frac{{\;\mathrm{weight}}_{\mathrm{ij}}-M{\left(t\right)}_{j}}{\mathrm{Std}.{\mathrm{Dev}}_{j}}$$

(3) $${\mathrm{BAZ}}_{\mathrm{ij}}=\frac{{\;\mathrm{BMI}}_{\mathrm{ij}}-M{\left(t\right)}_{j}}{\mathrm{Std}.{\mathrm{Dev}}_{j}}$$

where ${\mathrm{HAZ}}_{\mathrm{ij}}$, ${\mathrm{WAZ}}_{\mathrm{ij}}$, and ${\mathrm{BAZ}}_{\mathrm{ij}}$ were the HAZ, WAZ, and BAZ scores for child i in group j, which was defined by gender; age, ${\mathrm{height}}_{\mathrm{ij}}$, ${\mathrm{weight}}_{\mathrm{ij}}$, ${\mathrm{and}\;\mathrm{BMI}}_{\mathrm{ij}}$ were the height, weight, and BMI indexes for child i in group j; $M{\left(t\right)}_{j}$ was the median height, weight, and BMI index for the reference child group of the same gender and age; and $\mathrm{Std}.{\mathrm{Dev}}_{j}$ was the standard height, weight, and BMI index deviations in reference group j. The median and the standard height, weight, and BMI index deviations for the reference group were published by the World Health Organization (WHO) and used as the reference standards.

#### 3.2.2. Independent Variables

The independent variables were shown as a multiplicative variable in the DID method used in this paper. Due to China’s special Hukou policy, once born, each person is assigned a Hukou type (either an agricultural (rural) Hukou or a non-agricultural (urban) Hukou) based on place of birth and lineage [28]. HAGC subsidies identify whether households belong to urban or rural areas through the Hukou. First, because HAGC subsidies only benefited rural households, the dummy Rural variable indicated whether a child’s household belonged to an agricultural (rural) Hukou: Rural_i_ = 1, otherwise, Rural_i_ = 0. Second, the dummy Post variable indicated whether the survey year was in 2009 or later because the HAGC policy was widely implemented in 2009. If the survey year was 2009 or later: Post = 1, otherwise, Post = 0. The multiplicative variable, Rural * Post, indicated whether a child’s household was covered by the HAGC policy.

#### 3.2.3. Control Variables

As in previous studies, the main control variables were those considered to influence child health: individual child characteristics, household-level characteristics, and parental characteristics. For example, the individual child characteristics were their age and education [29,30]. Having health insurance was also considered a covariate that could potentially impact child health [11]. The household-level characteristics were household income and basic household health facility ownership [31,32]. The parental characteristics were parental time allocation [17] and parental health and education [33,34]. Table 1 shows the variables and associated definitions.

### 3.3. Estimation Strategy: Difference-in-Differences

To identify the causal relationships between appliance ownership and child health, the exogenous price variations generated by the “Home Appliances Going to the Countryside” (HAGC) policy were leveraged. As the HAGC policy is a national strategic plan that cannot be controlled by each region, its implementation was regarded as a quasi-natural experiment; therefore, the impact of household technology could be studied by comparing the changes in the health status of the rural and urban children, before and after the HAGC policy implementation.

To compare the changes in the health status of children that resulted from the HAGC policy implementation, the following was estimated:

(4) $${\mathrm{HEALTH}}_{\mathrm{it}}={\;\alpha }_{0}+\alpha_{1}{\mathrm{Rural}}_{\mathrm{it}}{*\mathrm{Post}}_{\mathrm{it}}+\beta_{1}X_{\mathrm{it}}+\varepsilon_{\mathrm{it}}$$

where ${\mathrm{HEALTH}}_{\mathrm{it}}$ was the health status of the children, in which subscript i was child i and subscript t was the year; ${\mathrm{Rural}}_{\mathrm{it}}$ denoted whether a child’s household belonged to a rural area (Rural = 1 meant the household belonged to the rural area; otherwise, Rural = 0); ${\mathrm{Post}}_{\mathrm{it}}$ = 1 was whether the survey year was in 2009 or later with ${\mathrm{Post}}_{\mathrm{it}}$ = 0 indicating that the survey year was before 2009; $\alpha_{1}$ was the household technology effect on the child’s health status; $X_{\mathrm{it}}$ was the control variable set affecting the child health status factor; and $\varepsilon_{\mathrm{it}}$ was the residual.

To obtain a consistent estimate for $\alpha_{1}$, the underlying assumption in Equation (4) was that the control group and the treatment group trends before the HAGC policy implementation were parallel. As in Beck et al. [35] the following equation was used:

(5) $$\begin{array}{ll} {\mathrm{HEALTH}}_{\mathrm{it}\;}=\; & \alpha_{0}+\gamma_{1}{\mathrm{Rural}}_{i,t-3}\;{*\;\mathrm{Post}}_{i,t-3}\\ & +\gamma_{2}{\mathrm{Rural}}_{i,t-2}\;{*\;\mathrm{Post}}_{i,t-2}+\ldots +\gamma_{6}{\mathrm{Rural}}_{i,t+2}\;{*\;\mathrm{Post}}_{i,t+2}+\beta_{1}X_{\mathrm{it}}+\varepsilon_{\mathrm{it}} \end{array}$$

where ${\mathrm{Rural}}_{i,t\pm n}\;{*\;\mathrm{Post}}_{i,t\pm n}$, respectively, represented the dummy variables for n years before and after the HAGC policy implementation. Taking the year of the approved HAGC policy as the boundary, this paper investigated the changing child health status trends in the three years before the HAGC policy implementation and the two years after the HAGC policy implementation. If the coefficients for policy, ${\mathrm{Rural}}_{i,t-n}\;{*\;\mathrm{Post}}_{i,t-n}$, were not significant, this indicated that the treatment and the control groups had parallel trends, and if the policy coefficients, ${\mathrm{Rural}}_{i,t+n}\;{*\;\mathrm{Post}}_{i,t+n}$, were not significant, this indicated that the driving effect of the HAGC policy was obvious.

The HAGC policy was first implemented in December 2007; therefore, the policy effect was not evident until 2008. The 2009 CHNS was conducted in July and August 2009 and reflected the situation in 2008. Therefore, the 2009 CHNS survey data only reflected the HAGC policy implementation in the pilot provinces (Shandong and Henan). After 2009, the HAGC policy covered all Chinese provinces, with the CHNS in 2011 reflecting the situation in 2010. Therefore, the CHNS data in 2011 and after were affected by the HAGC policy. To test the benchmark regression robustness (Equation (4)), the PSM-DID method was applied to estimate the household technology effects on child health by comparing the child health statuses in the pilot and non-pilot provinces, before and after 2009. The PSM-DID model estimates were determined using the following equation:

(6) $$\begin{array}{l} {\mathrm{HEALTH}}_{\mathrm{it}}\\ ={\;\alpha }_{0}+\gamma_{1}{\mathrm{Rural}}_{i,t-3}\;{*\;\mathrm{Post}}_{i,t-3}\\ +\gamma_{2}{\mathrm{Rural}}_{i,t-2}\;{*\;\mathrm{Post}}_{i,t-2}+\ldots +\gamma_{6}{\mathrm{Rural}}_{i,t+2}\;{*\;\mathrm{Post}}_{i,t+2}+\beta_{1}X_{\mathrm{it}}+\varepsilon_{\mathrm{it}} \end{array}$$

(7) $$p\left(X\right)=\;\mathrm{Pr} [D\;=\;1]$$

(8) $${\mathrm{HEALTH}}_{\mathrm{it}}={\;\alpha }_{0}+\alpha_{1}{\mathrm{Pilot}}_{\mathrm{it}}\;{*\;\mathrm{After}}_{\mathrm{it}}+\beta_{1}X_{\mathrm{it}}+\varepsilon_{\mathrm{it}}$$

where D represents all the cities including both treatment and control groups; X represented the control variables; ${\mathrm{Pilot}}_{\mathrm{it}}$ was whether or not the provinces where the children lived were pilot provinces (${\mathrm{Pilot}}_{\mathrm{it}}$ = 1 meant the province was a pilot province; otherwise, ${\mathrm{Pilot}}_{\mathrm{it}}$ = 0); ${\mathrm{After}}_{\mathrm{it}}$ = 1 indicated 2009 (after all provinces had been covered by the HAGC policy); and ${\mathrm{After}}_{\mathrm{it}}$ = 0 indicated 2006.

## 4. Results

### 4.1. Summary of Statistics

The summary of statistics for the key variables are shown in Table 2. After dropping the respondents with missing key explanatory variable information, the final analysis sample was an unbalanced panel data set with 5085 respondents comprising 2731 rural children and 2354 urban children.

**Figure 4:**
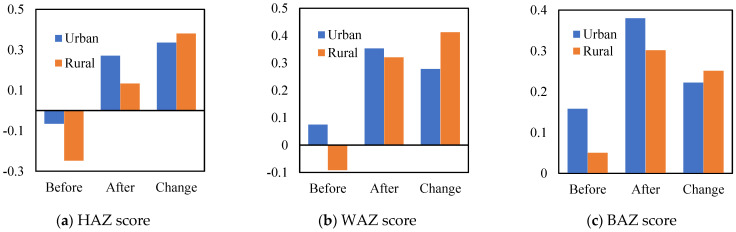
Changes in child health after the HAGC policy.

The average child health status in the rural areas was much lower than in the urban areas. The changes in the children’s health status in the rural and urban areas were compared before and after the HAGC policy. As shown in Figure 4, the health status of each index had a significant increase in the rural areas after the HAGC policy implementation relative to the rise in the urban areas. The average age of all samples was 9.6 years and only about 53% of children had medical insurance. The average child sleep time in the urban samples was higher than that in the rural samples, and as expected, the education levels of the children and parents in the urban areas was generally higher than in the rural areas. The examination of the path variables revealed that after the HAGC policy was implemented, the parents’ child-care time, appliance adoption rate, and nutrients all increased and that this was more significant in the rural areas than in the urban areas.

### 4.2. Baseline Results

The estimated benchmark regression results (Equation (4)) are given in Table 3 (Only a brief model variable table is provided here. Please refer to the Supplementary Materials for the complete model variable table), in which columns (1), (2), and (3), respectively, show the impact of household technology on the HAZ, WAZ, and BAZ scores. The estimated coefficients for Rural ∗ Post were significantly positive for all health indexes, and the benchmark regression results showed that household technology had had a significant impact on child health improvements. Specifically, the HAGC policy implementation improved the HAZ score with a coefficient of 0.059, which was statistically significant at the 5% level; improved the WAZ score with a coefficient of 0.083, which was statistically significant at the 1% level; and improved the BAZ score with a coefficient of 0.069, which was statistically significant at the 5% level. The HAZ, WAZ, and BAZ scores in the HAGC policy coverage area were, respectively, 5.9%, 8.3%, and 6.9% higher on average than in the HAGC policy non-coverage areas. This finding was consistent with Chen, et al.’s (2015) finding on the effect of household technology on adult health.

The examination of the control variables found that age and child health were closely related. The higher the age, the poorer the child’s health, which may have been because as a child grows, the greater the social impediments to equal health rights [36]. However, the higher the child’s education, the higher were the child’s HAZ and WAZ scores, as was also found in previous research [37]. Having medical insurance also had a significant improvement effect on the HAZ and WAZ scores, with coefficients of 0.109 and 0.073. This finding was consistent with [11], which found that public medical insurance reduced the possibility of children being stunted.

Child sleep time and child health status had a negative correlation. Previous research has found a bidirectional relationship between sleep and health [38]. Having a flush toilet significantly increased the child’s HAZ, WAZ, and BAZ scores, which was consistent with [5]. Of the parent characteristics, whether the parents smoked and their education level both significantly affected the child health status, which had also been found in previous research [34,39].

### 4.3. Test for Parallel Trends Assumption

A basic assumption of the DID estimation strategy is that without the intervention, the trends in the treatment group results would be the same as in the control group; that is, if the HAGC policy had had no external effect, the child health development trends in both the treatment and control groups would be parallel. Therefore, a parallel trend test was conducted to assess this assumption.

**Figure 5:**
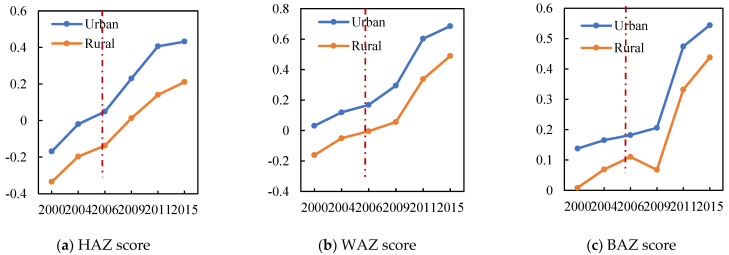
Child health status changes after the HAGC policy.

**Figure 6:**
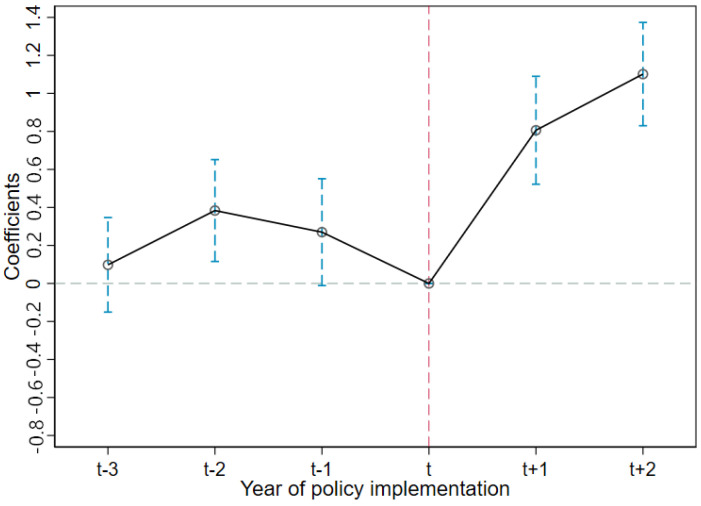
Effects of household technology on HAZ scores for the years before and after the treatment.

First, the changing child health trends were plotted. As shown in Figure 5, the health trends for the control and treatment groups were the same before the HAGC policy implementation. To further verify this parallel trend, Equation (5) was estimated and the coefficients for the effects of ${\mathrm{Rural}}_{i,t\pm n}*{\mathrm{Post}}_{i,t\pm n}$ on the child HAZ scores were plotted at 95% confidence intervals (see Figure 6).

As shown in Figure 6, before the HAGC policy implementation, the coefficient for ${\mathrm{Rural}}_{i,t\pm n}{*\mathrm{Post}}_{i,t\pm n}$ was not significant, that is, there were no significant differences in the HAZ score trends between the treatment and control groups. After the HAGC policy implementation, the coefficient was significantly positive, and this positive driving effect was evident in the whole sample period and had certain sustainability. Therefore, the treatment and control groups had no significant differences before the HAGC policy implementation.

### 4.4. Placebo Test

To further verify whether unobservable factors led to result biases, a virtual HAGC policy implementation time was randomly assigned to a person as a placebo test, that is, a “false” HAGC policy variable was created, after which the benchmark model (Equation (4)) was estimated 500 times using the “false” HAGC policy variable, and the estimated coefficients for the effects of ${\mathrm{Rural}}_{\mathrm{it}}{*\mathrm{Post}}_{\mathrm{it}}$ on the child HAZ score were stored.

**Figure 7:**
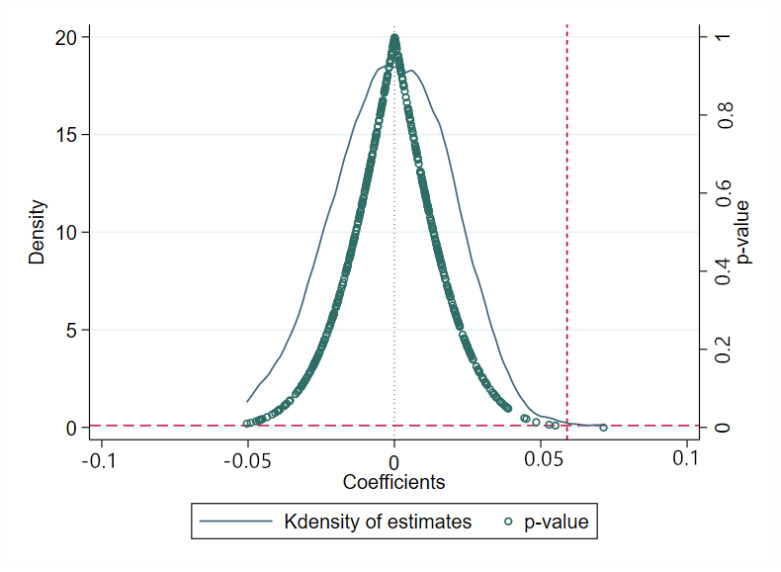
Household technology and child health: placebo test.

Figure 7 shows the empirical cumulative distribution function and density for the estimated coefficients for ${\mathrm{Rural}}_{\mathrm{it}}{*\mathrm{Post}}_{\mathrm{it}}$ impacts on the child HAZ scores. The estimated coefficients for the ${\mathrm{Rural}}_{\mathrm{it}}{*\mathrm{Post}}_{\mathrm{it}}$ placebo variable were distributed around zero; however, as the real estimated value of the benchmark was 0.059 (column (1) in Table 3), the ${\mathrm{Rural}}_{\mathrm{it}}{*\mathrm{Post}}_{\mathrm{it}}$ benchmark estimate coefficients for the HAGC policy variable lay outside the estimated virtual coefficient ranges, and the *p*-values were all above 0.1. This placebo test method has been widely used in DID processing in recent years [40,41].

### 4.5. Robustness Checks

To further verify the positive effect of household technology on child health status improvements, a robustness test was conducted. First, the robustness of the results was tested by constructing different dependent variables as child health status indicators. As the dependent variable measuring method, self-rated health, was also an indicator of individual health status, this was used to replace the health score. The results in column (1) in Table 4 show that the regression coefficients for ${\mathrm{Rural}}_{\mathrm{it}}{*\mathrm{Post}}_{\mathrm{it}}$ were still significantly positive, suggesting that household technology had a significant positive effect on child health status (Only a brief model variable table is provided here. Please refer to the Supplementary Materials for the complete model variable table).

To test the robustness of the DID method results, the PSM-DID approach was employed to estimate the child health status in pilot and non-pilot provinces before and after the policy implementation. As shown in columns (2) to (4) in Table 4, the regression coefficients for the HAGC policy variable remained significantly positive regardless of whether the DID method or the PSM-DID approach was used, indicating that the regression results showed that household technology significantly promoted child health and the benchmark regression estimation results were robust.

### 4.6. Heterogeneity Analysis

The gender and age impacts of household technology on child health status were then investigated. As Table 5 shows (Only a brief model variable table is provided here. Please refer to the Supplementary Materials for the complete model variable table), there were obvious differences. Specifically, the estimated coefficient for the independent variable was positive and statistically significant at a 10% level for girls, but was not statistically significant for boys, which was possibly because of the difference in the parents’ capital investment. In some Asian countries, such as China and India, boys are generally preferred to girls due to a traditional “son preference”; therefore, sons gain a greater share of intra-household resources [42,43], which means that the health status of girls could be more sensitive to the increased nutrition and parental care time resulting from the adoption of household appliances. As girls generally need to do more housework, the reduction in housework because of the new technologies could provide greater opportunities for girls to improve their physical health [4].

Table 6 shows the heterogeneous effects of household technology on child health at different ages (Only a brief model variable table is provided here. Please refer to the Supplementary Materials for the complete model variable table). The impact of household technology on the health status of young children and teenagers was obviously different. Specifically, the estimated coefficient for the independent variable was positive and statistically significant at a 5% level for teenagers, but was not statistically significant for younger children, that is, the health impact of household technology was more significant for teenagers. As intra-household resources tend to be more focused on young children, the young children generally have greater opportunities to access parental care and nutritious food. Therefore, the health status of young children would not be as sensitive to the increased nutrition and parental care resulting from household appliance adoption. As children grow, their parents’ time allocation changes, that is, as children become more independent, parents spend less time taking care of them and more time on other matters. Therefore, the health status of adolescents would be more sensitive to the increase in parental care time resulting from household technology adoption. As adolescents also need to do more housework, any reduction in housework time due to new family technology would give adolescents more time to improve their physical health.

### 4.7. Mechanism Analysis

The benchmark model results clearly showed that household technology enhanced child health. To disentangle the household technology effect between household technology adoption and child health status, an attempt was made to reveal the paths through which the household technology improved child health.

The first possible explanation is that household technology adoption affected parental time allocation. Previous studies have found that home technology adoption reduced individual housework time [44], and that parental time allocations affected child health [45]. To test this hypothesis, the HAGC policy implementation impact on parental time allocation was investigated, the results for which are shown in Table 7 (Only a brief model variable table is provided here. Please refer to the Supplementary Materials for the complete model variable table). Column (1) shows that the estimated coefficient for the independent variable was significantly negative, indicating that household technology significantly reduced the time needed for housework, and column (2) shows that the estimated coefficient for Rural ∗ Post was significantly positive, indicating that household technology significantly increased the time parents spent taking care of their children. Therefore, it could be surmised that after the HAGC policy was implemented, the parents’ housework times significantly decreased and the time they spent taking care of their children significantly increased, thereby proving that child health was impacted by household technology through the increase in parental time allocation.

The second possible explanation was that household technology affected child nutrition. It has been proven that household technology adoption can improve family nutrition [46,47]. Therefore, the HAGC policy implementation was taken as the proxy variable for household technology adoption, and the impact of the HAGC policy implementation on household nutrition was estimated, for which household energy, carbohydrate, oil, and protein intakes over three days were taken as the proxy variables. Columns (1) to (4) in Table 8 show that the estimated Rural ∗ Post coefficients were all significantly positive (Only a brief model variable table is provided here. Please refer to the Supplementary Materials for the complete model variable table), that is, the HAGC policy implementation increased household nutrition, possibly because the HAGC policy provided greater opportunities to access nutritious food.

To further clarify the impact of household technology adoption on child health, the specific impacts of the different home technologies were examined. Greenwood, et al. divided home appliances into time-saving appliances and time-consuming appliances [14]. Therefore, washing machines and refrigerators were considered to be time-saving appliances, and color TV was considered a time-consuming appliance. The refrigerator was considered a vital household technology to improve household nutrition. Table 9 reports the results for the different household technologies on child health (Only a brief model variable table is provided here. Please refer to the Supplementary Materials for the complete model variable table). As columns (1) to (4) show, the coefficients for washing machine adoption were not significant, and the coefficients for color TV and refrigerator adoption were significantly positive, which proved that the “parental time allocation” hypothesis was tenable. The estimated coefficient and statistical significance of the refrigerator were higher than for the washing machine, possibly because as the refrigerator can save time and promote child nutrition, the superposition of the two attributes resulted in a more significant impact of the refrigerator on child health.

## 5. Discussion

Although this paper proved that household technology adoption improved child health by changing parental time allocation and nutrition, there may be alternative explanations. For example, household technology adoption could also have adverse effects on child health. Specifically, the adoption of color TV could result in excessive TV watching and increase the risk of obesity [48]. Another explanation could be that household technology adoption may change household income distribution [49], by, for example, decreasing the expenditure on food and increasing the expenditure on energy.

This research had some limitations. First, although the CNHS is a continuous survey data set, the data from 2000 to 2015 was an unbalanced panel data set. Therefore, fixed-effect models could not be used to control the time-invariant individual heterogeneity. Second, due to data limitations, it was not possible to confirm how the household income distribution resulting from household technology adoption affected child health. However, this was beyond the scope of this study. These issues could be explored in further research when relevant information and resources are available.

## 6. Conclusions

In this paper, a DID strategy was used to identify the causal effects of household technology adoption on child health. With the household appliance adoption changes generated by the “Home Appliances Going to the Countryside” policy subsidies in China as the identification path, the rural and urban differences in child health before and after the implementation of the HAGC policy were examined. It was found that household appliance adoption had significant impacts on child health. More specifically, the HAGC policy increased the HAZ, WAZ, and BAZ scores by 5.9%, 8.3%, and 6.9%, respectively.

However, there was heterogeneity found in the impact of household technology on child health. The gender heterogeneity analysis found that the impact was significantly stronger for girls than boys and the age heterogeneity analysis found that the impact was significantly stronger for adolescents than for young children. To clarify the underlying mechanisms of these impacts, the parental time allocation and family nutrition paths resulting from household technology adoption were examined, with both being found to have positive impacts on child health. These findings can imply that policy makers could provide reasonable subsidies for household appliances to improve the health of children in developing countries. However, we still need to be cautious when generalizing our results to include the overall effects of household technology, because both the initial health status, as well as the socioeconomic status, of the Chinese rural population are quite different from those in more developed countries. As a result, the effects of home appliances on health outcomes may not be the same in other countries.

## Figures and Tables

**Table 1:** Variables and definitions.

Variable	Abbreviation	Description
**Dependent variable**		
HAZ score	HAZ	Continuous variable
WAZ score	WAZ	Continuous variable
BAZ score	BAZ	Continuous variable
Self-rated health	SRH	1 = Poor;2 = Fair;3 = Good;4 = Excellent
**Independent variables**		
Whether a child’s household was in a rural area	Rural	1 = rural area; otherwise, 0
Whether the survey year was 2009 or later	Post	1 = 2009 or later; otherwise 0
**Control variables**		
Age in years	Age	Continuous variable
Child education in years	Education	Continuous variable
Medical insurance	Insurance	1 = with access to Insurance; otherwise, 0
Sleep time in hours	Sleep time	Continuous variable
Flushing toilet availability	Toilet	1 = available flush toilet; 0 = otherwise
Tap water availability	Water	1 = available tap water; 0 = otherwise
Household income	Ln(income)	Continuous variable, inflated to Chinese CNY in 2015
Parents’ housework time in hours	P_H time	Continuous variable
Do parents smoke	P_Smoke	1 = at least one parent smoked; 0 = otherwise
Parental average education in years	P_Education	Continuous variable
**Path variables**		
Parents’ child-care time in hours	P_C time	Continuous variable
Color TV ownership	Color TV	1 = Own Color TV; 0 = otherwise
Washing machine ownership	Washing machine	1 = Own Washing machine; 0 = otherwise
Refrigerator ownership	Refrigerator	1 = Own Refrigerator; 0 = otherwise
Average three-day energy intake	Kcal	Continuous variable
Average three-day carbohydrate intake	Carbohydrate	Continuous variable
Average three-day fat intake	Fat	Continuous variable
Average three-day protein intake	Protein	Continuous variable

**Table 2:** Summary of statistics for the crucial variables.

	Before				After			
	Rural		Urban		Rural		Urban	
	Mean	SD	Mean	SD	Mean	SD	Mean	SD
**Dependent variable**								
HAZ	−0.25	0.92	−0.06	0.95	0.13	1.02	0.27	1.00
WAZ	−0.09	0.73	0.10	0.84	0.32	1.15	0.35	1.12
BAZ	0.05	0.70	0.16	0.81	0.30	1.14	0.38	1.20
SRH	2.05	1.24	2.13	1.37	2.34	1.74	2.23	1.40
**Control variables**								
Age	10.13	4.84	10.99	4.95	8.34	4.95	9.39	5.07
Education	9.52	5.40	12.15	5.81	11.73	13.02	18.00	6.31
Insurance	0.23	0.42	0.25	0.43	0.90	0.30	0.88	0.33
Sleep time	9.51	1.74	9.07	1.71	9.49	1.80	9.21	1.83
Toilet	0.26	0.44	0.64	0.48	0.57	0.50	0.86	0.34
Water	0.60	0.49	0.89	0.31	0.79	0.41	0.96	0.20
Ln(income)	9.67	0.92	9.99	0.98	10.52	1.08	10.75	1.16
P_H time	81.32	63.47	104.82	81.34	140.78	104.55	184.97	132.54
P_Smoke	0.01	0.11	0.01	0.10	0.00	0.05	0.01	0.10
P_Education	18.53	6.04	21.41	6.28	21.20	5.73	25.15	5.82
**Path variables**								
P_C time	10.11	44.41	12.08	48.18	21.97	42.00	20.15	44.60
Color TV	0.75	0.43	0.89	0.31	0.98	0.13	0.99	0.09
Washing machine	0.55	0.50	0.74	0.44	0.84	0.36	0.92	0.27
Refrigerator	0.31	0.46	0.62	0.48	0.78	0.42	0.90	0.31
Kcal	1727.92	672.65	1811.72	682.24	1517.73	619.60	1543.99	609.51
Carbohydrate	264.10	107.48	245.85	100.12	210.87	89.78	190.52	85.17
Fat	51.68	33.79	65.49	36.32	53.24	34.59	61.50	32.30
Protein	51.47	21.76	59.47	27.85	48.56	21.11	56.67	24.22
Observations	1.423		1.216		1.308		1.138	

**Table 3:** Benchmark regression estimation results for the effect of household technology on child health.

Variables	(1)	(2)	(3)
	HAZ	WAZ	BAZ
Rural × Post	0.059 **	0.083 ***	0.069 **
	(0.029)	(0.030)	(0.031)
Age	−0.086 ***	−0.064 ***	−0.025 ***
	(0.007)	(0.008)	(0.008)
Education	0.042 ***	0.023 ***	0.002
	(0.005)	(0.005)	(0.005)
Insurance	0.109 ***	0.073 **	0.024
	(0.030)	(0.032)	(0.032)
Sleep time	−0.064 ***	−0.060 ***	−0.029 **
	(0.011)	(0.012)	(0.012)
Water	0.008	0.010	0.012
	(0.034)	(0.036)	(0.037)
Toilet	0.165 ***	0.179 ***	0.116 ***
	(0.031)	(0.032)	(0.033)
Ln(income)	0.021 *	0.013	0.007
	(0.012)	(0.013)	(0.013)
P_H time	0.000	0.000 *	0.000
	(0.000)	(0.000)	(0.000)
P_Smoke	−0.342 ***	0.020	−0.290 **
	(0.126)	(0.132)	(0.136)
P_Education	0.018 ***	0.018 ***	0.012 ***
	(0.003)	(0.003)	(0.003)
Constant	0.100	0.373 **	0.287
	(0.176)	(0.185)	(0.189)
R-squared	0.081	0.061	0.023
Observations	5.085	5.085	5.085

Standard errors in parentheses; *** *p* < 0.01, ** *p* < 0.05, * *p* < 0.1.

**Table 4:** Robustness checks for different indicators and estimation methods.

	Alternative Health	Alternative Model (PSM–DID)		
	(1)	(2)	(3)	(4)
	SRH	HAZ	WAZ	BAZ
Rural × Post	0.141 ***			
	(0.051)			
Pilot × After		0.099 *	0.087 *	0.167 ***
		(0.051)	(0.046)	(0.056)
Constant	2.985 ***	−0.010	0.100 ***	0.282 ***
	(0.318)	(0.029)	(0.027)	(0.031)
Control variables	Y	Y	Y	Y
R-squared	0.011	0.140	0.002	0.811
Observations	4.797	1.562	1.562	1.540

Standard errors in parentheses; *** *p* < 0.01, * *p* < 0.1.

**Table 5:** Household technology and child health status: gender heterogeneity.

	Boys			Girls		
	(1)	(2)	(3)	(4)	(5)	(6)
	HAZ	WAZ	BAZ	HAZ	WAZ	BAZ
Rural × Post	0.035	0.066	0.052	0.077 *	0.093 **	0.078 *
	(0.039)	(0.041)	(0.042)	(0.043)	(0.045)	(0.046)
Constant	0.001	0.108	−0.015	0.278	0.689 **	0.638 **
	(0.238)	(0.250)	(0.255)	(0.264)	(0.276)	(0.284)
Control variables	Y	Y	Y	Y	Y	Y
R-squared	0.080	0.066	0.029	0.084	0.061	0.023
Observations	2.716	2.716	2.716	2.369	2.369	2.369

Standard errors in parentheses; ** *p* < 0.05, * *p* < 0.1.

**Table 6:** Household technology and child health status: age heterogeneity.

	Age < 12			Age > 12		
	(1)	(2)	(3)	(4)	(5)	(6)
	HAZ	WAZ	BAZ	HAZ	WAZ	BAZ
Rural × Post	0.044	0.055	0.052	0.108 **	0.145 ***	0.110 **
	(0.041)	(0.042)	(0.043)	(0.047)	(0.049)	(0.050)
Constant	−0.190	0.010	−0.035	−1.225 ***	−0.385	0.128
	(0.248)	(0.258)	(0.259)	(0.286)	(0.301)	(0.307)
Control variables	Y	Y	Y	Y	Y	Y
R-squared	0.058	0.054	0.025	0.073	0.039	0.012
Observations	2.564	2.564	2.564	2.099	2.099	2.099

Standard errors in parentheses; *** *p* < 0.01, ** *p* < 0.05.

**Table 7:** Mechanism analysis: impact of the household technology on parental time allocation.

	(1)	(2)
	P_H Time	P_C Time
Rural × Post	−13.854 **	5.949 ***
	(6.643)	(0.465)
Constant	124.753 ***	6.705 ***
	(2.291)	(0.259)
Control variables	Y	Y
R-squared	0.587	0.022
Observations	5.085	5.085

Standard errors in parentheses; *** *p* < 0.01, ** *p* < 0.05.

**Table 8:** Mechanism analysis: the impact of the household technology on nutrition intake.

	(1)	(2)	(3)	(4)
	Kcal	Carbohydrate	Fat	Protein
Rural × Post	143.138 ***	21.570 ***	5.605 **	14.667 ***
	(46.808)	(7.015)	(2.711)	(0.968)
Constant	1,628.123 ***	233.022 ***	54.160 ***	49.036 ***
	(12.483)	(1.871)	(0.723)	(0.344)
Control variables	Y	Y	Y	Y
R-squared	0.635	0.640	0.573	0.593
Observations	5.085	5.085	5.085	5.085

Standard errors in parentheses; *** *p* < 0.01, ** *p* < 0.05.

**Table 9:** Mechanism analysis: impact of household technology on nutrition intake.

	(1)	(2)	(3)
	HAZ	HAZ	BAZ
TV_color	0.050	−0.037	−0.048
	(0.060)	(0.063)	(0.064)
Washing_machine	0.131 ***	0.084 **	0.017 *
	(0.036)	(0.037)	(0.038)
Refrigerator	0.199 ***	0.259 ***	0.216 ***
	(0.033)	(0.035)	(0.036)
Constant	0.131	0.495 ***	0.407 **
	(0.180)	(0.189)	(0.195)
Control variables	Y	Y	Y
R-squared	0.094	0.075	0.100
Observations	5085	5085	5085

Standard errors in parentheses; *** *p* < 0.01, ** *p* < 0.05, * *p* < 0.1.

## Data Availability

Not applicable.

## Supplementary Materials

The following supporting information can be downloaded at: https://www.mdpi.com/article/10.3390/ijerph191911976/s1, Complete model variable table.
