# Supplementary material for: The Effect of Household Technology on Child Health: Evidence from China’s “Home Appliances Going to the Countryside” Policy
Source: Int J Environ Res Public Health. 2022 Sep 22;19(19):11976. doi: 10.3390/ijerph191911976 (PMC9565273; doi:10.3390/ijerph191911976)
Supplement: Supplementary file 1 [file ijerph-19-11976-s001.zip › ijerph-1826136-supplementary.pdf]

Supplementary Materials

Complete model variable table

Table S1. Benchmark regression estimation results for household technology on child health (Correspond to Table 3 in article).

VARIABLES	(1)<br>HAZ	(2)<br>WAZ	(3)<br>BAZ
Rural ∗ Post	0.059**	0.083***	0.069**
	(0.029)	(0.030)	(0.031)
Rural	0.032*	0.016**	0.033*
	(0.019)	(0.013)	(0.021)
Post	0.059**	0.083***	0.069**
	(0.029)	(0.030)	(0.031)
Age	-0.086***	-0.064***	-0.025***
	(0.007)	(0.008)	(0.008)
Education	0.042***	0.023***	0.002
	(0.005)	(0.005)	(0.005)
Insurance	0.109***	0.073**	0.024
	(0.030)	(0.032)	(0.032)
Sleep time	-0.064***	-0.060***	-0.029**
	(0.011)	(0.012)	(0.012)
Water	0.008	0.010	0.012
	(0.034)	(0.036)	(0.037)
Toilet	0.165***	0.179***	0.116***
	(0.031)	(0.032)	(0.033)
Ln(income)	0.021*	0.013	0.007
	(0.012)	(0.013)	(0.013)
P_H time	0.000	0.000*	0.000
	(0.000)	(0.000)	(0.000)
P_Smoke	-0.342***	0.020	-0.290**
	(0.126)	(0.132)	(0.136)
P_Education	0.018***	0.018***	0.012***
	(0.003)	(0.003)	(0.003)
Constant	0.100	0.373**	0.287
	(0.176)	(0.185)	(0.189)
R-squared	0.081	0.061	0.023
Observations	5,085	5,085	5,085

Standard errors in parentheses; *** p<0.01, ** p<0.05, * p<0.1.

Table S2. Robustness checks for different indicators and estimation methods (Correspond to Table 4 in article).

	Alternative health	Alternative model (PSM-DID)		
	(1)<br>SRH	(2)<br>HAZ	(3)<br>WAZ	(4)<br>BAZ
Rural ∗ Post	0.141***			
	(0.051)			
Pilot ∗ After		0.099*	0.087*	0.167***
		(0.051)	(0.046)	(0.056)
Age	-0.016	-0.101***	-0.077***	-0.030***
	(0.014)	(0.009)	(0.010)	(0.010)
Education	-0.014	0.058***	0.033***	0.003
	(0.008)	(0.006)	(0.006)	(0.006)
Insurance	0.013	0.095***	0.119***	0.088***
	(0.054)	(0.032)	(0.033)	(0.034)
Sleep time	-0.028	-0.101***	-0.103***	-0.057***
	(0.021)	(0.014)	(0.015)	(0.015)
Water	-0.120**	-0.114***	-0.042	0.017
	(0.061)	(0.042)	(0.043)	(0.044)

Toilet	0.084	0.017	0.005	-0.001
	(0.054)	(0.035)	(0.036)	(0.037)
Ln(income)	-0.006	0.065***	0.066***	0.038**
	(0.022)	(0.015)	(0.016)	(0.016)
P_H time	-0.000	0.001***	0.001***	0.000***
	(0.000)	(0.000)	(0.000)	(0.000)
P_Smoke	0.191	-0.079	0.190	0.322**
	(0.233)	(0.142)	(0.148)	(0.150)
P_Education	-0.003	0.041***	0.038***	0.024***
	(0.004)	(0.003)	(0.003)	(0.003)
Constant	2.985***	-0.010	0.100***	0.282***
	(0.318)	(0.029)	(0.027)	(0.031)
R-squared	0.011	0.140	0.002	0.811
Observations	4,797	1,562	1,562	1540

Standard errors in parentheses; *** p<0.01, ** p<0.05, * p<0.1.

Table S3. Household technology and child health status: gender heterogeneity (Correspond to Table 5 in article).

	Boys			Girls		
	(1) HAZ	(2) WAZ	(3) BAZ	(4) HAZ	(5) WAZ	(6) BAZ
Rural * Post	0.035	0.066	0.052	0.077*	0.093**	0.078*
	(0.039)	(0.041)	(0.042)	(0.043)	(0.045)	(0.046)
Age	-0.084***	-0.050***	-0.003	-0.091***	-0.080***	-0.051***
	(0.010)	(0.010)	(0.011)	(0.011)	(0.012)	(0.012)
Education	0.044***	0.014**	-0.011*	0.042***	0.034***	0.018**
	(0.006)	(0.006)	(0.007)	(0.007)	(0.007)	(0.007)
Insurance	0.123***	0.100**	0.053	0.093**	0.043	-0.005
	(0.042)	(0.044)	(0.045)	(0.044)	(0.046)	(0.047)
Sleep time	-0.066***	-0.061***	-0.030*	-0.067***	-0.063***	-0.033*
	(0.015)	(0.016)	(0.017)	(0.017)	(0.018)	(0.019)
Water	0.004	-0.009	-0.017	0.020	0.042	0.056
	(0.046)	(0.049)	(0.050)	(0.051)	(0.053)	(0.055)
Toilet	0.164***	0.171***	0.113**	0.163***	0.175***	0.102**
	(0.042)	(0.044)	(0.045)	(0.045)	(0.047)	(0.048)
Ln(income)	0.036**	0.037**	0.027	0.002	-0.015	-0.015
	(0.017)	(0.018)	(0.018)	(0.018)	(0.019)	(0.019)
P_H time	0.000	0.000	0.000	0.000	0.000**	0.000*
	(0.000)	(0.000)	(0.000)	(0.000)	(0.000)	(0.000)
P_Smoke	-0.508**	-0.109	0.222	-0.233	0.107	0.348**
	(0.217)	(0.228)	(0.233)	(0.156)	(0.163)	(0.168)
P_Education	0.016***	0.021***	0.017***	0.021***	0.015***	0.006
	(0.003)	(0.004)	(0.004)	(0.004)	(0.004)	(0.004)
Constant	0.001	0.108	-0.015	0.278	0.689**	0.638**
	(0.238)	(0.250)	(0.255)	(0.264)	(0.276)	(0.284)
R-squared	0.080	0.066	0.029	0.084	0.061	0.023
Observations	2,716	2,716	2,716	2,369	2,369	2,369

Standard errors in parentheses; *** p<0.01, ** p<0.05, * p<0.1.

Table S4. Household technology and child health status: age heterogeneity (Correspond to Table 6 in article).

	Age<12			Age>12		
	(1) HAZ	(2) WAZ	(3) BAZ	(4) HAZ	(5) WAZ	(6) BAZ
Rural * Post	0.044	0.055	0.052	0.108**	0.145***	0.110**
	(0.041)	(0.042)	(0.043)	(0.047)	(0.049)	(0.050)
Education	0.021***	0.011**	0.003	0.026***	0.006	-0.005
	(0.005)	(0.005)	(0.005)	(0.006)	(0.007)	(0.007)
Insurance	0.153***	0.048	-0.038	0.051	0.066	0.046

	(0.045)	(0.047)	(0.048)	(0.044)	(0.047)	(0.048)
Sleep time	-0.083***	-0.072***	-0.030*	-0.050***	-0.042**	-0.019
	(0.017)	(0.018)	(0.018)	(0.017)	(0.018)	(0.018)
Water	0.017	-0.016	-0.020	-0.001	0.068	0.075
	(0.049)	(0.051)	(0.051)	(0.054)	(0.057)	(0.058)
Toilet	0.168***	0.220***	0.164***	0.122**	0.146***	0.104**
	(0.043)	(0.045)	(0.045)	(0.048)	(0.051)	(0.052)
Ln(income)	0.018	0.012	0.010	0.047**	0.029	0.009
	(0.017)	(0.018)	(0.018)	(0.020)	(0.021)	(0.022)
P_H time	0.000*	0.000**	0.000	-0.000	0.000	0.000
	(0.000)	(0.000)	(0.000)	(0.000)	(0.000)	(0.000)
P_Smoke	-0.297	0.026	0.327	-0.513***	-0.192	0.065
	(0.217)	(0.225)	(0.227)	(0.170)	(0.179)	(0.183)
P_Education	0.016***	0.022***	0.017***	0.022***	0.012***	0.000
	(0.004)	(0.004)	(0.004)	(0.004)	(0.004)	(0.004)
Constant	-0.190	0.010	-0.035	-1.225***	-0.385	0.128
	(0.248)	(0.258)	(0.259)	(0.286)	(0.301)	(0.307)
R-squared	0.058	0.054	0.025	0.073	0.039	0.012
Observations	2,564	2,564	2,564	2,099	2,099	2,099

Standard errors in parentheses; *** p<0.01, ** p<0.05, * p<0.1.

Table S5. Mechanism analysis: impact of the household technology on parental time allocation (Correspond to Table 7 in article).

	(1)<br>**P_H time**	(2)<br>**P_C time**
Rural * Post	-13.854**	5.949***
	(6.643)	(0.465)
Insurance	26.744***	5.831***
	(2.278)	(1.961)
Water	4.421*	2.484
	(2.576)	(2.130)
Toilet	9.033***	-2.160
	(2.370)	(1.934)
Ln(income)	-1.087	0.193
	(1.004)	(0.828)
P_Smoke	3.317	-12.374
	(10.559)	(16.540)
P_Education	2.601***	0.092
	(0.179)	(0.167)
Constant	124.753***	6.705***
	(2.291)	(0.259)
R-squared	0.587	0.022
Observations	5,085	5,085

Standard errors in parentheses; *** p<0.01, ** p<0.05, * p<0.1.

Table S6. Mechanism analysis: the impact of the household technology on nutrition intake (Correspond to Table 8 in article).

	(1)<br>**Kcal**	(2)<br>**Carbohydrate**	(3)<br>**Fat**	(4)<br>**Protein**
Rural * Post	143.138***	21.570***	5.605**	14.667***
	(46.808)	(7.015)	(2.711)	(0.968)
Age	54.107***	9.356***	1.077***	1.674***
	(5.630)	(0.858)	(0.323)	(0.207)
Education	10.196***	0.569	0.765***	0.291**
	(3.542)	(0.539)	(0.204)	(0.130)
Insurance	-38.679*	-17.055***	2.959**	1.275*
	(20.555)	(3.112)	(1.190)	(0.754)
Sleep time	11.243	2.458*	0.171	-0.191
	(8.655)	(1.312)	(0.500)	(0.318)
Water	-31.139	-15.517***	3.359**	0.387
	(24.782)	(3.795)	(1.410)	(0.914)
Toilet	50.316**	-11.442***	8.741***	5.231***

	(21.743)	(3.331)	(1.237)	(0.802)
Ln(income)	22.675**	-2.340*	2.653***	1.810***
	(9.255)	(1.410)	(0.531)	(0.340)
P_H time	-0.027	-0.027**	0.005	0.008**
	(0.090)	(0.014)	(0.005)	(0.003)
P_Smoke	-146.497*	-20.319	-4.578	-4.569
	(88.518)	(13.529)	(5.058)	(3.262)
P_Education	1.954	-0.966***	0.521***	0.293***
	(1.893)	(0.291)	(0.107)	(0.070)
Constant	1,628.123***	233.022***	54.160***	49.036***
	(12.483)	(1.871)	(0.723)	(0.344)
Control variables	Y	Y	Y	Y
R-squared	0.635	0.640	0.573	0.593
Observations	5,085	5,085	5,085	5,085

Standard errors in parentheses; *** p<0.01, ** p<0.05, * p<0.1.

Table S7. Mechanism analysis: impact of household technology on nutrition intake (Correspond to Table 9 in article).

	**(1)**<br>**HAZ**	**(2)**<br>**HAZ**	**(3)**<br>**BAZ**
TV_color	0.050	-0.037	-0.048
	(0.060)	(0.063)	(0.064)
Washing_machine	0.131***	0.084**	0.017*
	(0.036)	(0.037)	(0.038)
Refrigerator	0.199***	0.259***	0.216***
	(0.033)	(0.035)	(0.036)
Age	-0.083***	-0.062***	-0.024***
	(0.007)	(0.008)	(0.008)
Education	0.041***	0.022***	0.001
	(0.004)	(0.005)	(0.005)
Insurance	0.088***	0.062**	0.021
	(0.028)	(0.029)	(0.030)
Sleep time	-0.060***	-0.055***	-0.026**
	(0.011)	(0.012)	(0.012)
Water	-0.036	-0.033	-0.017
	(0.034)	(0.036)	(0.037)
Toilet	0.093***	0.101***	0.059*
	(0.032)	(0.033)	(0.034)
Ln(income)	0.008	0.001	-0.001
	(0.012)	(0.013)	(0.013)
P_H time	0.000	0.000*	0.000
	(0.000)	(0.000)	(0.000)
P_Smoke	-0.300**	0.066	0.322**
	(0.126)	(0.132)	(0.135)
P_Education	0.013***	0.013***	0.008***
	(0.003)	(0.003)	(0.003)
Constant	0.131	0.495***	0.407**
	(0.180)	(0.189)	(0.195)
R-squared	0.094	0.075	0.100
Observations	5,085	5,085	5,085

Standard errors in parentheses; *** p<0.01, ** p<0.05, * p<0.1.
